# Sexual, dating, and bullying-related forms of violence targeting LGBTQ+ youth among a population-based sample of California high school students

**DOI:** 10.1038/s41598-026-48315-7

**Published:** 2026-04-28

**Authors:** Sabrina C. Boyce, Ricardo Vera Monroy, Emma Jackson, Riley Saham, Jay G. Silverman

**Affiliations:** 1 Community Health Sciences, School of Public Health, University of California, Berkeley, 2121 Berkeley Way #6130, Berkeley, CA USA; 2 Department of Social, Behavioral and Population Sciences, Celia S. Weatherhead School of Public Health and Tropical Medicine, Tulane University, New Orleans, LA USA

**Keywords:** LGBTQ+ youth, Violence, Bullying, Sexual violence, Gender minority youth, Health care, Psychology, Psychology, Risk factors

## Abstract

LGBTQ+ youth experience inequitably high risk for bullying, sexual violence (SV), and dating violence (DV), disproportionately disrupting their social, educational, financial, and health opportunities. LGBTQ+ youth may even be targeted for distinct forms of violence based on their LGBTQ+ identity (e.g., unwanted sexual contact to “prove” one’s gender or sexual orientation), yet little is known from population-based studies. This study provides prevalences for a range of ways LGBTQ+ youth are specifically targeted for bullying, SV, and DV using data from a population-based sample of 9th and 11th grade LGBTQ+ youth from 30 public schools in California (*N* = 2,284) in 2021–2022. Generalized estimating equations with robust standard errors were used to estimate the association between LGBTQ+ youths’ experiences of LGBTQ+-targeted violence and mental health, academic performance, and school connection. Results indicate that these forms of violence are prevalent, especially among transgender boys and nonbinary youth, and are associated with significantly higher risk for depressive symptoms, suicidality, school absenteeism, worse academic grades, lower school connection, and lower perceived safety while at school. This evidence calls for continued research to better understand the unique ways LGBTQ+ youth experience SV, DV, and bullying to ensure LGBTQ+ youth have access to a safe and supportive education.

## Introduction

LGBTQ+ youth (including but not limited to lesbian, gay, bisexual, transgender, nonbinary people ages 13–18 years) experience inequitably high risk for violence, including bullying (i.e., unwanted aggressive behavior by another youth or group of youths)^1^, sexual violence (i.e., sexual activity when consent is not obtained or freely given, SV)^2^ and dating violence (i.e., abuse or aggression that occurs in a romantic relationship, DV)^3,4^. In the U.S., where one in every four high school youth report a LGBTQ+ identity, 29% of LGBTQ+ youth [vs. 16% of cisgender heterosexual (i.e., non-LGBTQ+) youth] report bullying, 20% (vs. 8.5% of non-LGBTQ+ youth) report SV, and 18% (vs. 7.5% of non-LGBTQ+ youth) report DV victimization in the past 12 months^5^. SV, DV, and/or bullying victimization during adolescence, a key sensitive period in human development, is associated with a negative life-long sequelae of poor health and well-being^6^, including poor mental health^7,8^ and suicidality^9,10^, lower educational attainment^11,12^, revictimization^13,14^, and substance use^15–17^. Consistent with minority stress theory^18^ and demonstrated by systematic evidence, LGBTQ+ youth often experience even worse violence-related health outcomes than non-LGBTQ+ victims due to the accumulation of chronic discrimination-related stress^7–9,18,19^.

The disproportionate burden of bullying, SV, and DV experienced by LGBTQ+ youth is likely due, in large part, to social and structural marginalization^18,20^. At the societal-level of the social ecology, LGBTQ+ youth experience social marginalization that is driven by hetero- and cis-sexism, wherein expansive gender and/or sexual presentation is judged as inferior for deviating from binary cisgender and heterosexual gender norms in the larger society^21,22^. This marginalization of LGBTQ+ identities manifests across the entire social ecology, including at the interpersonal-level in the form of interpersonal violence, microaggressions, and discrimination against LGBTQ+ people. Discrimination-based violence against LGBTQ+ individuals, which has also been described as identity abuse, bias-based or identity-based bullying, can include but is not limited to the use of heterosexist/transphobic language, harassment or aggression perceived to be related to one’s identity, social pressure to conform to binary cisgender and heterosexual gender norms, and application of harmful stereotypes of LGBTQ+ people^22,23^. Social marginalization creates an environment of heightened vulnerability to violence for LGBTQ+ youth^20,24,25^. Moreover, according to theories of intersectionality, people who have intersecting marginalized identities (e.g., racially minoritized LGBTQ+ youth) experience overlapping systems of oppression within the social hierarchy, and thus, are often particularly targeted for structural marginalization and violence, including SV and DV^26–28^.

Most previous quantitative research on the distinct ways LGBTQ+ youth are targeted for violence has been limited to the use of a single measure of perceived bias-based bullying based on LGBTQ+ identity. For example, a population-based study of students in 27 Northeastern high schools found 40% of sexual minority and 53% of gender expansive youth reported bias-based bullying^29^. While an important initial step in documenting violence targeting LGBTQ+ youth, this single measure fails to capture many of the forms of violence targeted at LGBTQ+ youth, particularly those that are related to SV and DV, and/or experiences that may not be considered bullying. The Trevor Project, a national nonprofit supporting LGBTQ+ youth mental health, conducts the only identified quantitative studies that measure other specific forms of LGBTQ+-targeted violence, including one measure of SV, using online survey data collected from a large convenience sample of LGBTQ+ young people ages 13 to 24 in the U.S. Based on survey data from 2024, 23% of LGBTQ+ young people reported they had been physically threatened or harmed due to their LGBTQ+ identity in the past year, 32% were verbally harassed, and 9% experienced unwanted sexual contact because of their perceived LGBTQ+ identity^30^. More population-based research clarifying the ways LGBTQ+ youth are targeted for SV, DV, and bullying is needed.

More specifically, research on the ways LGBTQ+ youth are targeted for SV and DV, forms of SV/DV that are not as often experienced by heterosexual cisgender youth, are severely understudied. Emerging research, primarily qualitative and from LGBTQ+ adults, suggests that LGBTQ+ individuals may be targeted for SV due to negative and hypersexualized stereotypes of their identities^24,31,32^. Among LGBTQ+ adults, forms of DV that target LGBTQ+ identities have been found to include threatening to “out” a romantic partner^33^ and fetishizing LGBTQ+ identities as exotic^34,35^, particularly for transgender^34^ and bisexual individuals^36,37^. Measures for LGBTQ+ adults, including the Identity Abuse scale^38^, the Transgender IPV Scale^39^, and the Gay and Bisexual Men IPV scale^40^, offer some of the first quantitative survey measures that include some of the ways LGBTQ+ people are targeted for SV/DV, yet none have been adapted for use with LGBTQ+ youth populations. Moreover, while the negative health and well-being consequences of forms of SV/DV measured in the general population are well established, no research to date has assessed whether the health consequences of these LGBTQ+-specific forms of violence are similar. Such research is critical to understanding the specific prevention and support needs of LGBTQ+ youth and to understand how these experiences contribute to the disproportionate burden of poor health and well-being among LGBTQ+ individuals.

This study aims to begin to fill this gap by offering the first prevalence estimates for a wide range of ways LGBTQ+ youth may be targeted for bullying, SV, and DV in a population-based sample of LGBTQ+ youth. Moreover, we aim to assess the extent to which experiencing these identity-based forms of violence are consequential to LGBTQ+ youth, and gender expansive (GE; including but not limited to youth who identified as transgender boy or girl, nonbinary, agender, or gender exploring) youth in particular, health and well-being, including mental health, school engagement and connectedness, and perceived safety at school. We hypothesize that violence targeting LGBTQ + and GE identities will be common among LGBTQ + and GE youth, respectively, and that they will be associated with increased risk for negative health and academic outcomes.

## Methods

### Data source

The California Healthy Kids Survey (CHKS) is a voluntary, anonymous survey administered to students in California public schools. This widely used survey consists of a core module on student attitudes, behaviors, and well-being, with options of including custom modules on various topics. Districts that elect to participate in CHKS administer surveys to a census sample of all 9th and 11th grade high school students, typically biannually. The online survey instrument is administered during one class session by school staff and data are collected and stored securely online by WestEd.

An additional 10-minute custom module for secondary students was developed by the research team to assess attitudes, behaviors, and social norms related to sexual and dating violence (SV/DV) prevention. The custom module also includes expansive measures of sexual orientation and gender identity and a new, exploratory measure on LGBTQ+-targeted forms of violence that only LGBTQ+-identifying youth received. The module was developed for the purposes of an evaluation study of a SV/DV prevention program and based on the research team’s expertise on SV/DV, validated measures, consultation with a youth research advisory board (RAB) made up of 7–10 diverse youth, with over 50% of the group identifying as LGBTQ+, and with a California-based RAB made up of LGBTQ+ youth service providers across California. The survey was pilot tested with a group of 24 youth attending a public high school in Southern California, which included both LGBTQ + and heterosexual cisgender youth. Data were obtained via a data use agreement (DUA) between researchers at the University of California, Berkeley, WestEd, and the California Department of Education (CDE).

Informed consent was obtained from parents/guardians of participants below 16 years of age and directly from participants 16 years or older. Informed assent was provided by participants below 16 years of age. No incentives were provided, as is typical with CHKS administration, and youth could refuse to respond to any survey question. This study’s protocol was approved by the University of California, San Diego and the University of California, Berkeley Institutional Review Boards.

### Sample

Through the CHKS mechanism, cross-sectional data were collected from 9,411 9th and 11th grade high school students, a census sample from one urban metropolitan school district (26 high schools) and four semi-rural school districts (four high schools) located across Southern and Central California that voluntarily participated in this custom module within the California Healthy Kids Survey (CHKS) during the 2021–2022 academic year. Five of the high schools within the sample were purposively recruited by the research team based on selection for the evaluation study; data are from baseline data collection. The other 25 schools voluntarily opted-in to implement the SV/DV prevention module. While not a representative sample of California youth, the sample is racially/ethnically diversity and includes urban, suburban, and semi-rural populations.

For this analysis, only 9th and 11th grade youth who received questions from the SV/DV violence exposure module and provided quality data across the whole survey were eligible for inclusion (*n* = 9,411). Furthermore, we excluded youth who preferred not to respond to the sexual and gender identity questions (*n* = 339), had poor-quality data across the sexual and gender identity measures (e.g., selected all sexual or gender identities; *n* = 124), or who were missing responses across all LGBTQ and gender expansive violence questions (*n* = 173). Lastly, youth had to identify with at least one expansive sexual orientation (i.e., selected at least one response option other than heterosexual) or one expansive gender (i.e., selected at least one response option other than cisgender girl or cisgender boy) to be included in the sample (*n* = 2,284 LGBTQ+ youth).

### Measures

#### Sexual orientation and gender identity (SOGI)

The custom module included one gender identity item and one sexual orientation “select all that apply” item, the former with ten response options and the latter with nine response options, each of which included a definition (Table 1). Self-describe text responses were coded by one person, a co-author who identifies as an LGBTQ+ young adult, with supervision from the first author. To allow youth to be counted within each identity they selected, a separate binary variable was made for each sexual orientation: heterosexual, lesbian/gay, bisexual, pansexual/omnisexual, asexual/demisexual, queer/LGBQ+, and exploring. Similarly for gender identity, separate binary variables were made for each of the following gender identities: cisgender boy, cisgender girl, nonbinary/gender fluid (henceforth, nonbinary), transgender boy, and transgender girl, gender nonconforming (which includes individuals who selected gender nonconforming, agender, demigirl, demiboy, or self-described an expansive gender identity), and gender exploring.

**Table 1:** Sexual orientation and gender identity definitions^a^.

**Gender identities**	
Cisgender boy	A person who identifies as a man/boy and whose sex/gender assigned at birth was the same
Cisgender girl	A person who identifies as a woman/girl and whose sex/gender assigned at birth was the same
Transgender boy	A person who identifies as a man/boy and whose sex/gender assigned at birth was different
Transgender girl	A person who identifies as a woman/girl and whose sex/gender assigned at birth was different
Nonbinary	A person who does not identify as a man or a woman
Gender fluid	A person whose gender identity changes and fluctuates over time
Gender non-conforming/gender queer	A person who does not conform to gender norms/their gender exists outside of cisgender frameworks of gender
Demigirl/demiboy	A person who mostly identifies as a girl/boy regardless of their sex assigned at birth
Agender	A person who does not identify with the experience or idea of gender
Exploring/questioning	A person who has not labeled their gender identity
**Sexual Orientations**	
Straight/heterosexual	A person who is a man attracted to women, or a woman attracted to men
Lesbian/Gay	A person who is attracted to people of the same gender or sex
Bisexual	A person who is attracted to people of the same gender or sex and other genders or sexes
Pansexual	A person who is attracted to people of all genders and sexes
Queer	Umbrella term for expansive sexual identities not including heterosexual
Asexual	A person who experiences no or limited sexual attraction
Demisexual	A person who is attracted to people to whom they experience a strong emotional connection
Exploring/questioning	A person who has not labeled their sexual orientation

^a^Definitions were developed and used for the California Healthy Kids custom module implemented as part of this research and provided to respondents in the questionnaire.

These sexual orientation and gender identity categories were combined to make 36 intersectional SOGI binary variables. With guidance from our RAB and similarity in terms of risk for violence victimization, the cisgender queer boy group together with cisgender pansexual/omnisexual boy group were collapsed into one variable, as were transgender girls of all sexual identities and as were transgender boys of all sexual identities, so that cell sizes were large enough to be included in results, given our data use agreement requirement of at least 10 individuals in a given cell. These 36 SOGI variables, which allow participants to be included in all the identity groups they selected, were used for violence prevalence estimates^41^.

For regression analyses, which aimed to assess the risk these forms of violence pose to LGBTQ+ youth well-being, the full sample was used for violence targeting LGBTQ+ youth and the sample of GE youth (i.e., youth who selected any gender identity other than cisgender boy or girl) was used for violence targeting GE youth.

#### Violence targeting LGBTQ+ youth

Students who identified as LGBTQ+ were asked four questions assessing their experiences of LGBTQ+ youth-targeted violence. Specifically, they were asked whether in their lifetime the following ever happened: (1) Someone threatened to tell people about their sexual orientation or gender identity; (2) Someone forced them to participate in sexual behaviors to prove their gender or sexual identity was ‘real’; (3) Someone called them derogatory names that have to do with their LGBTQ+ identity; and (4) A dating or romantic partner prevented them from seeking support from other people within the LGBTQ+ community. Items were adapted from the *Identity Abuse* scale created for LGBTQ+ adults by a team of violence and LGBTQ+ youth scholars, reviewed by the youth RAB, and pilot tested, as described above regarding the development of the module^38^. Response options included, “Yes, this happened in the past 12 months”, “Yes, this happened but not in the past 12 months”, “No this never happened”, and “Prefer not to answer”. Anyone who selected one of the “yes” responses to experiencing at least one of these behaviors was categorized as having experienced violence targeting LGBTQ+ youth. If participants reported one of the two “yes” responses, they were also asked whether the person who did it to them was a dating partner or someone else. Anyone who selected a dating partner was included in the estimation of violence by a dating partner.

#### Violence targeting GE youth

Students who identified as a gender other than cisgender were asked three additional questions to assess their experiences of violence targeting their gender. Specifically, they were asked whether in their lifetime the following had occurred: (1) Someone forced them to do something that conflicted with their gender identity or to change their gender presentation; (2) Someone hid or destroyed property related to their gender transition or presentation, such as hormones, prosthetics, or binders; and (3) Someone attempted to touch them, or did touch them, to ‘figure out’ their gender. Items were adapted for youth from adult measures, the *Identity Abuse* scale^38^ and the *Transgender-specific Intimate Partner Violence (T-IPV)* scale, again by a team of violence and LGBTQ+ youth scholars, reviewed by the youth RAB, and pilot tested, as described above regarding the development of the module^39^. Response options included, “Yes, this happened in the past 12 months”, “Yes, this happened but not in the past 12 months”, “No this never happened”, and “Prefer not to answer”. Anyone who selected one of the “yes” responses to experiencing at least one of these behaviors was categorized as having experienced violence targeting GE youth (GE-targeted violence). If participants said “yes”, they were asked whether the person who did it to them was a dating partner or someone else to estimate violence perpetrated by a dating partner.

*Dependent variables* include measures of well-being across mental health, academic engagement, school connection selected by WestEd. Outcomes were assessed using either single-item measures or validated scales. For all scale-based outcomes, mean responses and standard deviations were calculated and treated as continuous variables. For all measures, higher values reflect a greater presence of the construct being measured (e.g., stronger school connectedness).

Mental health was assessed as depressive symptoms and suicidality. For *depressive symptoms*, students answered whether they had felt so sad or hopeless almost every day for two weeks or more that they stopped doing some usual activities in the past 12 months (Yes/No). For *suicidality*, they were asked if they had seriously considered attempting suicide in the past 12 months (Yes/No).

Academic engagement was assessed as self-reported academic performance and school absenteeism. Self-reported *academic performance* was assessed over the past 12 months, with response options ranging from “Mostly As” to “Mostly Fs”, with high scores reflecting better performance. *School absenteeism* was assessed by asking students how many full days they had missed school in the past 30 days (0 days, 1 day, 2 days, or 3 + days).

School connection was assessed as the students’ relationship with adults at school, having an adult at school with high expectations of them, connectedness to the school, and perceived safety. *Connectedness to a caring adult in school* (Cronbach’s alpha = 0.851; 4-point Likert scale; 4 items) was assessed as the extent to which a teacher or another adult at school, for example, really cares about them, notices when they’re not there, and listens when they have something to say (4 = Very Connected). *High expectations from a teacher/adult in school* (Cronbach’s alpha = 0.85; 4-point Likert scale; 3 items) was measured as the extent to which a teacher or another adult tells them when they do a good job, always wants them to do their best, and believes they will be a success (4 = Very Much True). *School Connectedness* (Cronbach’s alpha = 0.78; 5-point Likert scale; 5 items) was measured as degree of agreement with a sense of belonging, happiness, and connection to their school community (5 = Strongly Agree). Finally, *perceived lack of safety at school* (5-point Likert scale; 1 item) was assessed by “How safe do you feel when you are at school?” (5 = Very Unsafe).

*Covariates* were selected a priori based on a directed acyclic graph^42^, previous literature, and availability of individual-level data in the dataset to control for potential confounding factors. Covariates included student’s race/ethnicity, parent’s education level, and student’s grade level. The race/ethnicity item was a multiple-choice question, requiring recategorization for mutually exclusive categories. Participants who selected more than one racial/ethnic identities were categorized as multiracial, those who selected Hispanic/Latinx were categorized as Hispanic/Latinx, and those who did not select Hispanic and did not select more than one race were categorized into the racial group they selected. The multiracial, other, Native American/Alaskan Native, and Pacific Islander/Native Hawaiian were combined to ensure cell sizes were large enough for running regression analyses. Parental education levels were based on student reports and were recategorized into four groups: “High School Incomplete,” “High School Graduate,” “Attended or Graduated from College,” and “Don’t know.”

### Analysis

Descriptive statistics were conducted to assess overall prevalence of each form of violence and prevalence of these forms of violence across SOGI groups. Cell sizes smaller than 10 individuals were suppressed to ensure anonymity of participants, per the DUA. Descriptive statistics were also conducted to explore the relationships between experiencing these forms of violence and youth well-being (mental health, academic engagement, school connection) and to explore collinearity between experiences of violence and covariates. For binary outcomes, which were not rare (rare defined as < 10%), unadjusted and adjusted generalized estimating equation (GEE) Poisson models with robust (Huber sandwich) standard errors and clustering at the school-level were applied to estimate relative risk of mental health outcomes based on experiencing violence targeting LGBTQ+ youth. For continuous outcomes, unadjusted and adjusted GEE linear regression models with robust (Huber sandwich) standard errors and clustering at the school-level were applied. Models were run for each exposure-outcome combination, with exposures including lifetime experiences of (1) violence targeting LGBTQ+ youth among all LGBTQ+ youth and (2) violence targeting GE youth among all GE youth. Multivariable models controlled for parent’s education level, student’s grade level, and student’s race/ethnicity. Complete case analyses were conducted as missing data were less than 1% for all outcome variables. P-values less than 0.05 were considered significant.

#### Code availability

Models were implemented using R version 4.5.0^43^. Code for these analyses is available upon request from the Corresponding Author.

## Results

### Socio-demographics of the sample

In this population-based sample of 9,255 youth, 2,284 youth identified as LGBTQ + and had complete sexual orientation and gender identity (SOGI) data and data on LGBTQ+-related violence experiences, with 31.6% (*n* = 722) of those also identifying as GE (Tables 1 and 2). The largest subgroups among LGBTQ+ youth were bisexual cisgender girls (28.9%) and exploring cisgender girls (15.0%). The largest subgroups of GE youth identified as pansexual/omnisexual nonbinary (19.3%), bisexual nonbinary (16.3%), and transgender boys (14.7%). The sample is racially/ethnically diverse, with 39.0% identifying as Hispanic/Latino, 19.2% as Asian/Asian American, and 6.1% as Black/African American, and 9.2% as more than one race/ethnicity. Three out of five (60.7%) reported depressive symptoms in the past 12 months, 35.7% reported suicidality, 20.5% reported 3 + days of school absenteeism in the past 30 days, and 3.3% reported mostly Fs for the past 12 months’ grades. In this sample, the average score was 2.57 (out of 4) for connectedness to a caring adult, 2.92 (out of 4) for high expectations from a teacher/adult at school, 3.38 (out of 5) for school connectedness, 2.51 (out of 5) for perceived lack of safety at school.

**Table 2:** Sociodemographic Characteristics of LGBTQ+ Youth Sample (*n* = 2,284).

Sociodemographic Characteristics	LGBTQ+ Youth (*n* = 2,284)	GE Youth (*n* = 722)
	*n* (%)	*n* (%)
**Grade Level**		
9th Grade	1,143 (50.0)	391 (54.2)
11th Grade	1,141 (50.0)	331 (45.8)
**Race/Ethnicity**		
Non-Hispanic White	604 (26.5)	199 (27.6)
Hispanic or Latino	889 (39.0)	266 (36.9)
Non-Hispanic Asian or Asian American	439 (19.2)	129 (17.9)
Non-Hispanic Black or African American	139 (6.1)	47 (6.5)
Multiracial/Other	210 (9.2)	80 (11.1)
**Parental Education**		
College Graduate or Some College	1,513 (66.2)	486 (67.3)
High School Graduate	262 (11.5)	78 (10.8)
High School Incomplete	254 (11.1)	75 (10.4)
Don’t know	255 (11.2)	83 (11.5)
**Sexual & Gender Identity** ^a^		
Cisgender heterosexual boy	56 (2.5)	*
Cisgender gay boy	76 (3.3)	*
Cisgender bisexual boy	161 (7.0)	*
Cisgender queer/pansexual/omnisexual boy	62 (2.7)	15 (2.1)
Cisgender asexual/demisexual boy	61 (2.7)	*
Cisgender exploring boy	67 (2.9)	*
Cisgender heterosexual girl	103 (4.5)	*
Cisgender gay girl	121 (5.3)	21 (2.9)
Cisgender bisexual girl	660 (28.9)	44 (6.1)
Cisgender pansexual/omnisexual girl	198 (8.7)	29 (4.0)
Cisgender asexual/demisexual girl	156 (6.8)	22 (3.0)
Cisgender queer girl	135 (5.9)	23 (3.2)
Cisgender exploring girl	342 (15.0)	25 (3.5)
Transgender boy (Any sexual orientation)	106 (4.6)	106 (14.7)
Transgender girl (Any sexual orientation)	41 (1.8)	41 (5.7)
Nonbinary heterosexual	12 (0.5)	12 (1.7)
Nonbinary gay	73 (3.2)	73 (10.1)
Nonbinary bisexual	118 (5.2)	118 (16.3)
Nonbinary pansexual/omnisexual	139 (6.1)	139 (19.3)
Nonbinary asexual/demisexual	94 (4.1)	94 (13.0)
Nonbinary queer	80 (3.5)	80 (11.1)
Nonbinary exploring	44 (1.9)	44 (6.1)
Gender non-conforming heterosexual	*	*
Gender non-conforming gay	37 (1.6)	37 (5.1)
Gender non-conforming bisexual	56 (2.5)	56 (7.8)
Gender non-conforming pansexual/omnisexual	64 (2.8)	64 (8.9)
Gender non-conforming asexual/demisexual	57 (2.5)	57 (7.9)
Gender non-conforming queer	77 (3.4)	77 (10.7)
Gender non-conforming exploring	28 (1.2)	28 (3.9)
Gender exploring heterosexual	*	*
Gender exploring gay	32 (1.4)	32 (4.4)
Gender exploring bisexual	69 (3.0)	69 (9.6)
Gender exploring pansexual/omnisexual	55 (2.4)	55 (7.6)
Gender exploring asexual/demisexual	49 (2.1)	49 (6.8)
Gender exploring queer	72 (3.2)	72 (10.0)
Gender exploring sexually exploring	82 (3.6)	82 (11.4)

*GE* gender expansive, *LGBTQ+* lesbian, gay, bisexual, transgender, questioning.

*Cells with fewer than 10 individuals are suppressed to preserve anonymity based on data use agreement.

^a^Sexual and gender identity groups are not mutually exclusive, so there is overlap of individuals across groups based on responses to a check-all-that-apply question.

### Prevalence of LGBTQ+-targeted and GE-targeted violence

Among LGBTQ+ youth, 36.0% reported any violence targeting their LGBTQ+ identity, with 75% of gender exploring gay youth, 73% of gay gender non-conforming youth, 72.6% of gay nonbinary youth, 72.5% of nonbinary queer youth reporting LGBTQ+-targeted violence (Table 3). Of the four forms of violence targeting LGBTQ+ identities that were measured, verbal abuse related to LGBTQ+ identity was reported the most (32.9%), followed by threat of involuntary disclosure of one’s LGBTQ+ identity (15.1%). Forced sex to prove one’s LGBTQ+ identity was rare, with 4.8% LGBTQ+ youth reporting this experience, but was most common among youth who identified as gender non-conforming queer (17.6%) and pansexual/omnisexual (16.7%). Experiencing these forms of LGBTQ+-targeted violence by a dating partner was reported by 5.5% of LGBTQ+ youth, with the largest proportion reporting that a dating partner isolated them from their LGBTQ+ community (4.0%).

**Table 3:** Experiences of Violence Targeting LGBTQ+ Youth by Sexual Orientation and Gender Identity Groups among LGBTQ+ Identifying Youth (*n* = 2,284).

Sexual Orientation & Gender Identity	Any Violence Targeting LGBTQ+ Youth (Ever)	Threat of Involuntary Disclosure	Forced Sex to Prove Identity	Verbal Abuse Related to LGBTQ+ Identity	Isolation from LGBTQ+ Community
	n (%)	n (%)	n (%)	n (%)	n (%)
**All LGBTQ+ Youth**	*n* = 2284	*n* = 2217	*n* = 2227	*n* = 2210	*n* = 2217
Violence perpetrated by anyone	823 (36.0)	334 (15.1)	106 (4.8)	728 (32.9)	89 (4.0)
Violence perpetrated by a dating partner	125 (5.5)	17 (0.7)	18 (0.8)	24 (1.1)	89 (4.0)
**By Sexual Orientation & Gender Identity**					
Cisgender heterosexual boy	*	0 (0.0)	*	*	0 (0.0)
Cisgender gay boy	39 (51.3)	13 (17.3)	*	38 (50.0)	*
Cisgender bisexual boy	49 (30.4)	16 (10.1)	*	45 (28.1)	*
Cisgender queer/pansexual/omnisexual boy	27 (43.5)	*	*	26 (41.9)	*
Cisgender asexual/demisexual boy	*	0 (0.0)	*	*	0 (0.0)
Cisgender exploring boy	14 (20.9)	*	*	13 (19.4)	*
Cisgender heterosexual girl	*	*	*	*	*
Cisgender gay girl	55 (45.5)	24 (20.3)	*	52 (43.7)	*
Cisgender bisexual girl	198 (30.0)	75 (11.7)	16 (2.5)	164 (26.1)	18 (2.8)
Cisgender pansexual/omnisexual girl	80 (40.4)	28 (14.7)	10 (5.2)	68 (35.6)	12 (6.4)
Cisgender asexual/demisexual girl	39 (25.0)	20 (12.9)	*	32 (21.1)	*
Cisgender queer girl	60 (44.4)	23 (17.3)	*	53 (40.5)	*
Cisgender exploring girl	54 (15.8)	15 (4.5)	*	41 (12.3)	*
Transgender boy (Any sexual orientation)	67 (63.2)	30 (30.6)	11 (11.3)	66 (66.0)	14 (13.9)
Transgender girl (Any sexual orientation)	19 (46.3)	11 (27.5)	*	17 (44.7)	*
Nonbinary heterosexual	*	*	*	*	0 (0.0)
Nonbinary gay	53 (72.6)	30 (42.3)	*	51 (73.9)	10 (14.3)
Nonbinary bisexual	66 (55.9)	31 (27.9)	13 (11.7)	62 (55.4)	*
Nonbinary pansexual/omnisexual	89 (64.0)	46 (33.6)	12 (9.3)	82 (60.7)	15 (11.0)
Nonbinary asexual/demisexual	55 (58.5)	29 (31.5)	*	51 (55.4)	12 (12.9)
Nonbinary queer	58 (72.5)	28 (36.4)	*	52 (66.7)	11 (14.1)
Nonbinary exploring	30 (68.2)	12 (27.9)	*	28 (65.1)	*
Gender non-conforming heterosexual	*	*	0 (0.0)	*	0 (0.0)
Gender non-conforming gay	27 (73.0)	14 (38.9)	*	26 (72.2)	*
Gender non-conforming bisexual	36 (64.3)	15 (26.8)	*	36 (66.7)	*
Gender non-conforming pansexual/omnisexual	40 (62.5)	20 (31.7)	10 (16.7)	38 (61.3)	11 (17.2)
Gender non-conforming asexual/demisexual	30 (52.6)	15 (26.3)	*	29 (54.7)	*
Gender non-conforming queer	52 (67.5)	24 (32.9)	13 (17.6)	49 (64.5)	*
Gender non-conforming exploring	16 (57.1)	*	*	15 (53.6)	*
Gender exploring heterosexual	*	0 (0.0)	0 (0.0)	*	0 (0.0)
Gender exploring gay	24 (75.0)	14 (43.8)	*	24 (75.0)	*
Gender exploring bisexual	34 (49.3)	16 (23.9)	*	32 (49.2)	*
Gender exploring pansexual/omnisexual	29 (52.7)	11 (20.4)	*	28 (52.8)	*
Gender exploring asexual/demisexual	27 (55.1)	11 (22.9)	*	27 (56.3)	*
Gender exploring queer	45 (62.5)	19 (27.1)	10 (14.5)	43 (62.3)	*
Gender exploring sexually exploring	29 (35.4)	*	*	27 (33.3)	*

*LGBTQ+* lesbian, gay, bisexual, transgender, questioning.

*Cells with fewer than 10 individuals are suppressed to preserve anonymity based on data use agreement.

Among GE youth, 29.5% reported any violence targeting their GE identity, with gender non-conforming gay (56.5%) and transgender boys (53.6%) reporting the highest prevalences (Table 4). Of the three forms of violence targeting GE identities that were measured, being forced to do something against one’s gender identity was the most common (25.8%), followed by unwanted touching to “figure out” their gender (11.2%) and hiding or destroying gender transition tools (6.5%). Transgender boys (22.0%), nonbinary queer (21.9%), and gender non-conforming queer (21.3%) youth reported the highest prevalences of experiencing unwanted touching to “figure out” their gender identity and 13.0% of nonbinary pansexual/omnisexual youth reported having hormones, prosthetics, or binders hidden or destroyed. It was rare that these forms of violence were perpetrated by a dating partner, with only 1.7% reporting a dating partner as the perpetrator of any of these forms of violence.

**Table 4:** Experiences of Violence Targeting Gender Expansive Youth by Sexual and Gender Identity Group among Gender Expansive Identifying Youth (*n* = 583).

Sample	Any Violence Targeting GE Youth	Forced to Do Something Against Gender Identity	Hormones, Prosthetics, or Binders Hidden or Destroyed	Unwanted Touching to ‘Figure Out’ Gender Identity
	*n* (%)	*n* (%)	*n* (%)	*n* (%)
**All GE youth**	*n* = 583	*n* = 551	*n* = 570	*n* = 569
Violence perpetrated by anyone	172 (29.5)	142 (25.8)	37 (6.5)	64 (11.2)
Violence perpetrated by a dating partner	10 (1.7)	*	*	*
**By Sexual Orientation and Gender Identity**				
Transgender boy (Any sexual orientation)	52 (53.6)	46 (50.5)	10 (10.9)	20 (22.0)
Transgender girl (Any sexual orientation)	11 (28.2)	11 (30.6)	*	*
Nonbinary heterosexual	*	*	0 (0.0)	0 (0.0)
Nonbinary gay	22 (38.6)	20 (37.0)	*	10 (18.2)
Nonbinary bisexual	27 (29.0)	24 (27.9)	*	*
Nonbinary pansexual/omnisexual	43 (37.1)	34 (33.3)	15 (13.0)	17 (14.9)
Nonbinary asexual/demisexual	32 (38.6)	27 (35.5)	*	10 (12.0)
Nonbinary queer	32 (49.2)	29 (47.5)	*	14 (21.9)
Nonbinary exploring	12 (35.3)	10 (30.3)	*	*
Gender non-conforming heterosexual	*	*	0 (0.0)	0 (0.0)
Gender non-conforming gay	13 (56.5)	10 (45.5)	*	*
Gender non-conforming bisexual	13 (34.2)	12 (33.3)	*	*
Gender non-conforming pansexual/omnisexual	19 (35.2)	17 (33.3)	*	*
Gender non-conforming asexual/demisexual	16 (33.3)	15 (34.1)	*	*
Gender non-conforming queer	23 (37.7)	19 (31.7)	*	13 (21.3)
Gender non-conforming exploring	*	*	0 (0.0)	*
Gender exploring heterosexual	0 (0.0)	0 (0.0)	0 (0.0)	0 (0.0)
Gender exploring gay	*	*	*	*
Gender exploring bisexual	*	*	*	*
Gender exploring pansexual/omnisexual	*	*	*	*
Gender exploring asexual/demisexual	12 (35.3)	10 (31.3)	*	*
Gender exploring queer	14 (25.9)	11 (21.6)	*	*
Gender exploring sexually exploring	*	*	0 (0.0)	*

*GE* gender expansive.

*Cells with fewer than 10 individuals are suppressed to preserve anonymity based on data use agreement.

### Association of LGBTQ+-targeted violence with educational and wellbeing outcomes

Descriptive results of the relationship between LGBTQ+-targeted and GE-targeted violence and well-being outcomes are in Table 5. In unadjusted and adjusted models, we found that LGBTQ + and GE youth who experienced targeted violence experienced greater risk for poor mental health and well-being outcomes (Table 6). In adjusted models among LGBTQ+ youth, those who had experienced LGBTQ+-targeted violence had 52% greater risk of depressive symptoms in the past 12 months [adjusted relative risk (aRR): 1.52; 95% confidence intervals (CI): 1.44–1.60] and over two times the risk of experiencing suicidality (aRR: 2.09, 95% CI: 1.93–2.27), relative to LGBTQ+ youth who did not report LGBTQ+-targeted violence (Table 5). Additionally, youth who had experienced these forms of violence reported more school absenteeism (β: 0.23, 95% CI: 0.16–0.31) and perceived greater lack of safety at school (β: 0.41; 95% CI: 0.33, 0.49), comparted to their peers. They also experienced significantly less school connectedness (β: −0.29; 95% CI: −0.36, −0.22), had significantly lower academic performance (β: −0.20, 95% CI: −0.31, −0.09), as well as slightly lower perceived expectations from teachers/adults (β: −0.11; 95% CI: −0.17, −0.04) and slightly less connectedness to a caring adult (β: −0.09; 95% CI: −0.15, −0.02), relative to other LGBTQ+ peers.

**Table 5:** Well-being Outcomes Among LGBTQ+ Youth by Experience of Any LGBTQ+-Targeted Violence and Any GE-Targeted Violence.

	Violence Targeting LGBTQ+ Youth (Ever; *n* = 2262)		Violence Targeting GE Youth (Ever; *n* = 575)	
	Yes (*n* = 813)	No (*n* = 1449)	Yes (*n* = 171)	No (*n* = 404)
**Well-Being Outcomes — Categorical**				
	*n (%)*	*n (%)*	*n (%)*	*n (%)*
Depressive Symptoms (Past 12 mos.)	628 (77.3)	742 (51.3)	148 (86.5)	250 (62)
Suicidality (Past 12 mos.)	434 (53.5)	371 (25.7)	112 (65.5)	171 (42.5)
**Well-Being Outcomes — Continuous**				
	*Mean (SD)*	*Mean (SD)*	*Mean (SD)*	*Mean (SD)*
School Absenteeism — Past 30 days (0–3; higher = more absences)	1.26 (1.2)	1.01 (1.17)	1.34 (1.2)	1.09 (1.23)
Perceived Lack of Safety at School (1–5; higher = less safe)	2.77 (0.84)	2.36 (0.77)	2.98 (0.91)	2.61 (0.84)
Connectedness to a Caring Adult (1–4; higher = more connected)	2.52 (0.85)	2.6 (0.82)	2.5 (0.86)	2.53 (0.82)
High Expectations from Teacher/Adult (1–4; higher = higher expectations)	2.86 (0.83)	2.96 (0.8)	2.82 (0.85)	2.88 (0.79)
School Connectedness (1–5; higher = more connected)	3.19 (0.74)	3.48 (0.69)	3.07 (0.8)	3.32 (0.69)
Academic Performance (1–8; higher = better performance)	6.12 (1.94)	6.31 (1.82)	5.9 (1.99)	6.14 (1.88)

*GE* gender expansive, *LGBTQ+* lesbian, gay, bisexual, transgender, questioning.

Categorical outcomes reported as n (%) of respondents endorsing Yes on that item within each exposure group. Denominator is non-missing responses on that item within the exposure group.

Continuous outcomes reported as Mean (SD). Denominator is non-missing responses on that variable within the exposure group.

For School Absenteeism and Perceived Lack of Safety at School, higher values indicate worse outcomes. For all other continuous outcomes (Connectedness to a Caring Adult, High Expectations from Teacher/Adult, School Connectedness, Academic Performance), higher values indicate better outcomes.

**Table 6:** Associations Between Experiencing Any Violence Targeting LGBTQ+ Youth and Well-Being Outcomes.

		Violence Targeting LGBTQ+ Youth (Ever)			Violence Targeting GE Youth (Ever)		
		*N*	Unadjusted Est (95% CI)	Adjusted Est (95% CI)	*N*	Unadjusted Est (95% CI)	Adjusted Est (95% CI)
Mental Health							
Depressive Symptoms (Past 12 mos.)	RR	2258	1.51*** (1.42, 1.60)	1.52*** (1.44, 1.60)	574	1.40*** (1.29, 1.51)	1.40*** (1.29, 1.51)
Suicidality (Past 12 mos.)	RR	2252	2.08*** (1.90, 2.27)	2.09*** (1.93, 2.27)	573	1.54*** (1.33, 1.80)	1.50*** (1.26, 1.78)
**School Climate**							
School Absenteeism (Past 30 days)	β	2256	0.24*** (0.16, 0.32)	0.23*** (0.16, 0.31)	573	0.24** (0.09, 0.39)	0.27*** (0.12, 0.43)
Perceived Lack of Safety at School	β	2257	0.41*** (0.32, 0.49)	0.41*** (0.33, 0.49)	573	0.36*** (0.17, 0.54)	0.37*** (0.19, 0.54)
Connectedness to a Caring Adult	β	2258	−0.08** (−0.14, −0.02)	−0.09* (−0.15, −0.02)	575	−0.03 (−0.19, 0.13)	−0.04 (−0.21, 0.12)
High Expectations from Teacher/Adult	β	2259	−0.10*** (−0.16, −0.04)	−0.11*** (−0.17, −0.04)	575	−0.05 (−0.17, 0.08)	−0.07 (−0.19, 0.06)
School Connectedness	β	2262	−0.29*** (−0.36, −0.21)	−0.29*** (−0.36, −0.22)	575	−0.24** (−0.38, −0.09)	−0.25** (−0.40, −0.10)
**Academic**							
Academic Performance	β	2261	−0.18* (−0.31, −0.04)	−0.20*** (−0.31, −0.09)	575	−0.24 (−0.66, 0.17)	−0.33 (−0.71, 0.05)

*CI* confidence interval, *GE* gender expansive, *LGBTQ+* lesbian, gay, bisexual, transgender, questioning, *RR* relative risk.

*p <.05; **p <.01; ***p <.001.

RR = relative risk estimated using generalized estimating equation (GEE) Poisson models with a log link and robust (Huber sandwich) standard errors, clustered at the school level (K = 29–30 schools). β = mean difference estimated using GEE linear models with an identity link and robust standard errors, clustered at the school level. All models use an exchangeable working correlation structure.

Adjusted models control for parent’s education level, student’s grade level, and student’s race/ethnicity.

N reflects the analytic sample for each adjusted model (complete cases on outcome, exposure, and covariates). Unadjusted models may include additional respondents with complete exposure and outcome data but missing covariates.

For School Absenteeism and Perceived Lack of Safety at School, higher values indicate worse outcomes (positive estimates indicate greater absenteeism or less safety among those experiencing violence). For Connectedness to a Caring Adult, High Expectations from Teacher/Adult, School Connectedness, and Academic Performance, higher values indicate better outcomes (negative estimates indicate worse well-being among those experiencing violence).

In adjusted models among GE youth, GE youth who had experienced GE-targeted violence were at 40% higher risk of having depressive symptoms in the past 12 month (aRR: 1.40; 95% CI: 1.29–1.51) and at 50% higher risk of experiencing suicidality (aRR: 1.50, 95% CI: 1.26–1.78), relative to GE youth who had not experienced these forms of violence. GE youth who reported GE-targeted violence, on average, missed more school (β: 0.27, 95% CI: 0.12–0.43), perceived a greater lack of school safety (β: 0.37, 95% CI: 0.19–0.54), and felt less connected to school (β: − 0.25, 95% CI: − 0.40, − 0.10), relative to GE youth who did not report these forms of violence (Table 6). No significant association was found for experiencing GE-targeted violence with connectedness to a caring adult, expectations from teachers/adults, or academic performance among GE youth.

## Discussion

The current study found that about one in three LGBTQ+ youth experience LGBTQ+-targeted violence and about one in three GE youth experience GE-targeted violence. Unaccounted for in standard measurements of SV, this study found that more than one in ten GE youth reported having experienced unwanted touching by someone to “figure out” their gender identity and one in 20 LGBTQ+ youth have experienced forced sex to prove their LGBTQ+ identity. Results from this study offer some of the first population-based estimates of the distinct ways in which LGBTQ+ youth are targeted for bullying, sexual violence, and dating violence, beyond the forms of violence more typically measured and experienced by non-LGBTQ+ youth, helping to advance measurement and recognition of these types of LGBTQ+-targeted experiences among youth.

Moreover, this study provides evidence that LGBTQ+ youth experiencing violence targeting their LGBTQ+ identity experience elevated risk of poor mental health and academic outcomes, in addition to the already high risk experienced by LGBTQ+ youth^30^. In particular, this study provides evidence that youth who experience violence targeting their LGBTQ+ identity experienced significantly greater risk for depressive symptoms, suicidality, as well as more school absenteeism, worse academic grades, less school connection, and lower perceived safety while at school. These negative consequences are similar to the consequences of SV, DV, and bullying more typically measured and recognized in the general population^7–12^, yet these forms of violence and their consequences have been under-recognized in research, practice, or services related to violence prevention and support and disproportionately affect LGBTQ+ youth.

These findings build upon limited previous research on forms of violence experienced by LGBTQ+ youth. Compared to school-based studies that have assessed experiences of bullying related to LGBTQ+ identity, this study found a similarly high prevalence of verbal harassment related to one’s LGBTQ+ identity, a form of violence that may be captured in a measurement of bullying^29,44^. More closely aligned with measurement in the current study, The Trevor Project’s study using a national convenience sample of LGBTQ+ youth found similar prevalence estimates for verbal harassment, interference with gender expression, and unwanted sexual contact related to LGBTQ+ identity^30^. The current study adds to this literature by assessing for forms of violence these previous studies have not, including unwanted touching to “figure out” someone’s gender; hiding or destroying hormones, prosthetics, or binders; threat of involuntary disclosure of their LGBTQ+ identity; isolation from the LGBTQ+ community; and these forms of violence as perpetrated by a dating partner, using a population-based sample. This study helps expand the current understanding of how LGBTQ+ youth are targeted for violence. Across nearly all forms of violence included in this study, transgender boys and nonbinary youth who identify as gay, pansexual, or asexual/demisexual were especially targeted for violence. These findings are consistent with theories of intersectionality and minority stress theory that emphasize how individuals who are marginalized along multiple axes experience compounding minority stress, which includes heightened risk for discrimination-based violence^18,28,45^. These findings are also consistent with empirical research from national and California samples of youth that indicate gender expansive youth face the highest risk for SV, DV, and bullying, and have the highest risk for related negative health outcomes, like poor mental health and suicidality^1,2,4,46^. The current study provides violence prevalence estimates for a wide range of LGBTQ+ subgroups, rather than assuming all LGBTQ+ youth have similar experiences, to understand the variation of experiences and needs within the LGBTQ+ youth population.

These results should be considered in the context of several limitations. First, this sample is not representative of all California or U.S. youth and is, instead, from a convenience sample of 30 schools in southern and central California. The 30 schools did, however, provide a census sample (i.e., population-based sample) of all 9th and 11th grade students and together provide strong racial/ethnic and rural/urban diversity in the sample. Second, despite our large sample, there was still some data sparsity across SOGI categories, which motivated collapsing categories so that cell sizes remained large enough to be reportable per confidentiality agreements. This trade-off of obscuring some small SOGI groups by combining them in order to ensure we could include their data and experiences in the study was a decision made in partnership with our RABs. Third, these are cross-sectional data such that the causal direction between exposure to these forms of violence and the well-being outcomes cannot be established. All outcomes were assessed for the past 12 months or more recent and violence was a lifetime measure, allowing us to make some assumptions about the violence occurring before outcomes, but temporality is not certain. Fourth, while we sought to expand measurement of violence experiences to include ways LGBTQ+ youth may be targeted for violence, the measurement is preliminary and is not exhaustive in capturing all of the ways LGBTQ+ youth may be targeted and may include forms of violence also experienced by cisgender heterosexual youth who are perceived to be gender non-conforming. In addition to the expertise and pilot testing used to create this measure, more qualitative research is needed to identify all the ways LGBTQ+ youth are particularly targeted for violence and to further develop this measure. Moreover, this measurement used in this analysis was a lifetime assessment and did not capture frequency or severity of violence. Future research using past 12 month reports of these forms of violence and the frequency of these experiences would be useful for further describing the scope of the problem.

Despite these limitations, this study offers some of the first population-based evidence that LGBTQ+ youth experience distinct forms of SV, DV, and bullying that have received limited attention from researchers and practitioners. Additionally, this study estimates prevalences for 36 unique SOGI groups and allowed youth to identify with multiple SOGI groups. Estimates from this research suggest that these experiences are common and consequential among LGBTQ + and GE youth, including for their mental health and academic outcomes, contributing to the already high risk for poor mental health and academic engagement among LGBTQ+ youth. This evidence calls for continued research to better understand the ways LGBTQ+ youth experience SV, DV, and bullying to ensure violence prevention and support services meet the needs of the LGBTQ+ population, a population that already faces some of the highest risk for these forms of violence. Moreover, these results provide evidence of how LGBTQ+-targeted victimization during adolescence likely contributes to life-long social determinants of LGBTQ+ health, including education and physical and psychological safety.

## Data Availability

The data that support the findings of this study are available from WestEd California School Climate, Health, and Learning Surveys (CalSCHLS) Program ([calschls@wested.org](mailto: calschls@wested.org)) but restrictions apply to the availability of these data, which were used under license for the current study, and so are not publicly available. Data are however available from the corresponding authors upon reasonable request and with permission of WestEd and the California Department of Education (contact [calschls@wested.org](mailto: calschls@wested.org) to request access).
